# Virtual Reality “exergames”: A promising countermeasure to improve motivation and restorative effects during long duration spaceflight missions

**DOI:** 10.3389/fphys.2022.932425

**Published:** 2022-10-11

**Authors:** Nathan Keller, Richard S. Whittle, Neil McHenry, Adam Johnston, Colton Duncan, Lori Ploutz-Snyder, Gabriel G. De La Torre, Melinda Sheffield-Moore, Gregory Chamitoff, Ana Diaz-Artiles

**Affiliations:** ^1^ Department of Health and Kinesiology, Texas A&M University, College Station, TX, United States; ^2^ Department of Aerospace Engineering, Texas A&M University, College Station, TX, United States; ^3^ Movement Science Program, School of Kinesiology, University of Michigan, Ann Arbor, MI, United States; ^4^ Department of Psychology, University of Cadiz, Cadiz, Spain; ^5^ Department of Internal Medicine, The University of Texas Medical Branch, Galveston, TX, United States

**Keywords:** sprint protocol, HIIT (high intensity interval training), resistance exercise, aerobic exercise, biometric, exergaming

## Abstract

Long duration spaceflight missions will require novel exercise systems to protect astronaut crew from the detrimental effects of microgravity exposure. The SPRINT protocol is a novel and promising exercise prescription that combines aerobic and resistive training using a flywheel device, and it was successfully employed in a 70-day bed-rest study as well as onboard the International Space Station. Our team created a VR simulation to further augment the SPRINT protocol when using a flywheel ergometer training device (the Multi-Mode Exercise Device or M-MED). The simulation aspired to maximal realism in a virtual river setting while providing real-time biometric feedback on heart rate performance to subjects. In this pilot study, five healthy, male, physically-active subjects aged 35 ± 9.0 years old underwent 2 weeks of SPRINT protocol, either with or without the VR simulation. After a 1-month washout period, subjects returned for a subsequent 2 weeks in the opposite VR condition. We measured physiological and cognitive variables of stress, performance, and well-being. While physiological effects did not suggest much difference with the VR condition over 2 weeks, metrics of motivation, affect, and mood restoration showed detectable differences, or trended toward more positive outcomes than exercise without VR. These results provide evidence that a well-designed VR “exergaming” simulation with biometric feedback could be a beneficial addition to exercise prescriptions, especially if users are exposed to isolation and confinement.

## Introduction

Pushing the Frontier of human spaceflight will require ever-increasing mission durations that will, in turn, require novel and creative solutions to the bigger demands on mission resources. Physical exercise remains the primary countermeasure to mitigate the health and performance decrements in astronauts caused by exposure to altered gravity environment (Clément, 2017; Richter et al., 2017; Diaz-Artiles et al., 2019). Astronauts typically exercise for 2 hours a day, 6 days a week, when onboard the International Space Station (ISS) (Hackney et al., 2015). On ISS, astronauts enjoy a suite of exercise modalities, including a cycle ergometer, treadmill, and resistive device. Trans-lunar and planetary missions will not feature such generous volume and mass allotments for their exercise systems and therefore, these missions will require the development of a singular, more integrative device as well as highly efficient protocol prescriptions (Smitherman and Schnell, 2020). A comprehensive solution to these problems remains elusive, although VR has been suggested as a promising candidate (Solignac and Kuntz, 2015; Salamon et al., 2017).

Volume, mass, usability, ease of maintenance, and schedule constraints will ultimately inform the final design of a long-duration mission exercise system. The operational usage of the system is another aspect to consider, and it is here where novel technologies and techniques can be leveraged into the mission. Finally, given the durations involved in trans-planetary missions, it is reasonable to suggest that no single system will suffice for eliciting the positive physiological and psychological effects typically associated with long-term exercise habits. Thus, integrating elements that increase variability within the exercise system and its operation, could highly benefit crewmembers embarked on a long duration exploration mission.

**FIGURE 1:**
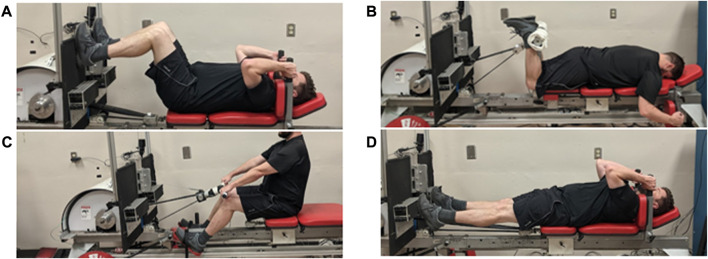
The Multi-Mode Exercise Device (M-MED) is capable of four modes of exercise. **(A)** Supine leg press. **(B)** Prone knee flexion. **(C)** Flywheel rowing ergometry. **(D)** Supine ankle plantarflexion.

The current project addresses this gap through the integration of different exercise modalities and the engagement of operational strategies intended to maximize the performance of exercise countermeasures. In particular, we leverage the SPRINT protocol, a duration/intensity-modulating exercise protocol successfully deployed in a 70-day bedrest study (Ploutz-Snyder et al., 2014, 2018) as well as onboard the ISS (English et al., 2020). The protocol features increased exercise intensity *via* high-intensity interval training, which reduces the required exercise time, thus liberating crew time for other tasks in their busy schedules. The convergence of Virtual Reality (VR) gaming with exercise, often called “exergaming,” is a promising technology to enhance well-being, enjoyment, and motivation while reducing negative stress and perceived exertion (Flores et al., 2008; Murray et al., 2016). The current investigation integrates both VR gaming and exercise using a prototype exercise device designed for the needs of astronaut crew in the spaceflight environment (Tesch et al., 2013; Cotter et al., 2015). This device is called the Multi-Mode Exercise Device (M-MED), a compact flywheel ergometer that accommodates four different exercise modes (see Figure 1): supine leg press (Panel A), prone knee flexion (B), flywheel rowing ergometry (C), and supine ankle plantarflexion (D). Additional resistance can be added as angular inertia in the latter three configurations *via* 2 kg steel plates slotted externally onto the flywheel’s main drive shaft. The capability to switch between cardiovascular and resistance training without meaningfully changing the volume and mass resource design requirements of the device marks a departure from the current state of the art onboard the ISS, where the crew utilize multiple devices to perform different exercise types, occupying most of the Tranquility module’s habitation volume. The M-MED delivers resistance loads to the same muscle groups as the devices on the ISS with the exception of the canonical “push” groups (pectoralis major, triceps brachii, deltoid group, *etc.*). As these latter groups are not weight-bearing, the effect of microgravity is not as pronounced and therefore, they are less critical targets for countermeasures (de Boer et al., 2008).

Given the complexity of integrating all of these aspects for the first time, it was prudent to conduct a pilot study to validate the integration of the SPRINT protocol, the VR intervention, and the M-MED training device, and to determine the best metrics for detecting differences due to the VR intervention. We therefore selected physiological and cognitive tools broadly in order to capture these differences (if they do exist) in participants with a similar health profile to astroanuts (i.e. mid-30’s, and physically fit). We expect that this preliminary work will provide a truly progressive step forward in the state-of-the-art of VR exergames countermeasures.

## Materials and methods

### Participants and oversight

Five male subjects were recruited according to the same criteria used in previous M-MED studies (Owerkowicz et al., 2016) (Cromwell et al., 2018): healthy subjects with a maximum oxygen uptake (VO_2Max_) of at least 30 ml/kg/min and isokinetic knee extensor strength of at least 2 N*m/kg of bodyweight. Subject age was 35.4 ± 9.0 years old (mean ± SD), and starting Body Mass Index (BMI) was 28.7 ± 5.96. Subjects received written and verbal reviews of the study protocol and they signed their informed consent. This protocol was approved by the Texas A&M Internal Review Board on human subjects under study number IRB 2019-0471 F.

### Testing modalities

A complete overview of the M-MED, the SPRINT protocol, and the VR scenario implemented in this study have been detailed previously (Keller et al., 2021).

Briefly, the M-MED, described earlier, was the exercise modality used in this study. Cardiovascular training was performed using a flywheel ergometer. Reconfiguring the M-MED device also allowed subjects to perform supine prone knee flexion, supine leg press, and supine ankle plantarflexion resistance exercises.

The SPRINT protocol required subjects to perform cardiovascular training 6 days/week (in our case, using the M-MED’s rowing ergometer configuration) and resistance training 3 days/week (*via* the remaining M-MED configurations described above) (Ploutz-Snyder et al., 2018). During cardiovascular training, subjects alternated among the following two options: 1) rowing continuously for 30 min at 75% of their heart rate based on their baseline VO_2Max_, or 2) performing high-intensity interval training (HIIT) exercise using rowing intervals of 30 s, 2 min, or 4 min at varying heart rate intensities (based on baseline VO_2Max_). Thus, in each HIIT training session, subjects performed one of the following three protocols: a) 8*30 s at maximal effort with 15 s of active rest, b) 6*2 min at the following heart rate intensities: 70%, 80%, 90%, 100%, 90%, and 80%, with 2 min of active rest, or c) 4*4 min at 85% heart rate intensity with 3 min of active rest. Each of the HIIT protocols was performed once a week. The weekly order of the HIIT workouts was randomized across subjects, but preserved for a given subject between conditions (VR vs No-VR). Resistance training was performed on the same days as continuous rowing with a gap of at least 4 hours between both types of training. Subjects performed the following lower leg exercises: supine leg presses, prone leg curls, and supine ankle plantarflexion. Resistance loads varied nonlinearly throughout the protocol, beginning with three sets of 10 repetitions, then increasing loads until the maximum possible angular resistance allowed by the flywheel, then increasing repetitions as needed throughout the workout until muscle failure or 20 repetitions (whichever came first).

**FIGURE 2:**
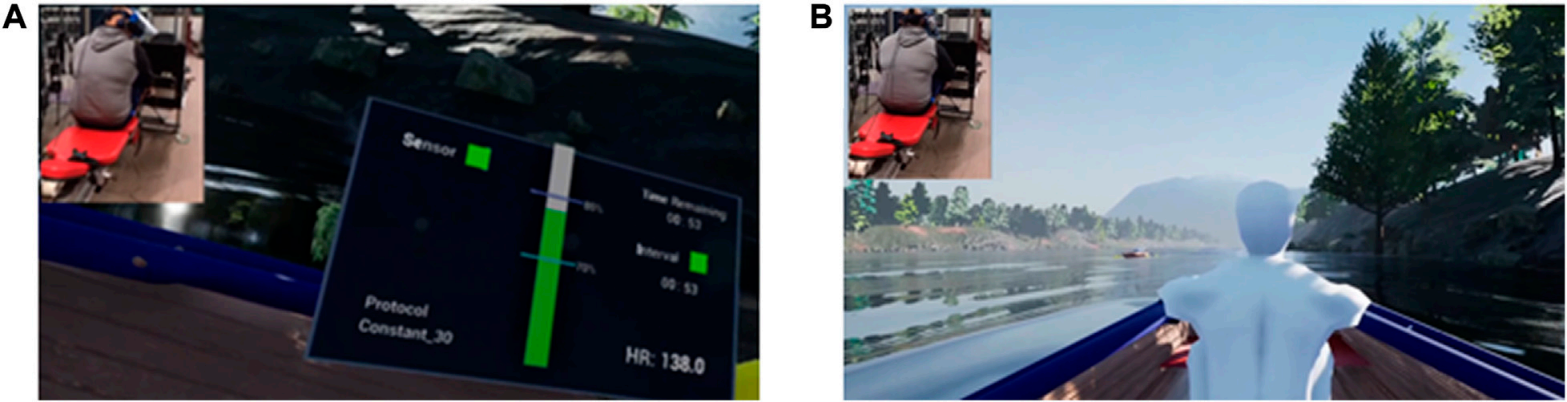
VR simulation with subject’s real-time recording inset. **(A)** subject viewing real-time readouts in a virtual display, including biometric heart rate data, time remaining in the protocol, sensor connection status, and protocol information. **(B)** subject viewing the position of the other virtual boat competitors located upstream. Note the vertical green bar on the virtual display in **(A)**, indicating successful attainment of target heart rate. This state corresponds to surpassing, or “winning against”, the virtual competitors as seen in **(B)**. If the subject’s heart rate became too low or too high with respect to the goal, the bar turns red and the competing boat gains velocity and ultimately passes the subject, unless they were able to re-attain goal heart rate.

A custom-made VR simulation was developed for this investigation. The simulation was integrated with the M-MED device and deployed during cardiovascular training (both continuous rowing and HIIT training). During these sessions, subjects were seated in a virtual boat with two virtual teammates pitted against a second boat of three virtual competitors. Both boats were situated in a river scene designed to seem as realistic as possible, including natural soundscape and oar-splashing audio components (synced to visual components). Audio was also delivered *via* the Vive headset’s onboard speakers, which occluded most external noises. Subjects’ heart rate was monitored *via* a chest-strap (Polar H10, Polar Electro 2020) and data were streamed into the simulation and presented to the subjects *via* a biometric display in their rowing boat. This display, shown in Figure 2A, showed the real-time quantitative heart rate as well as a qualitative vertical bar indicating whether the heart rate was within the expected limits (+/– 5% of the heart rate goal). In addition, if subjects were not maintaining their heart rate goal (i.e., heart rate became too low or too high with respect to the goal), the velocity of second boat increased in real time, exceeding the velocity of the subjects’ boat. Conversely, if the heart rate goal was successfully maintained, the velocity of the second boat fell just below the subjects’ boat velocity (see Figure 2B). The downstream distance between the two boats was limited to 10 m to prevent an uncompetitive runaway scenario. VR simulations were not utilized during the resistance training due to the short duration of these exercise sessions.

### Experimental design

**FIGURE 3:**
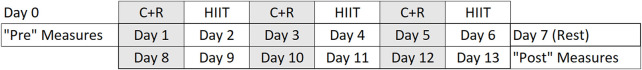
SPRINT protocol and timeline implemented in the study. Subjects exercised for 2 weeks in each one of the two experimental conditions (VR vs. No-VR). C + R indicates days of 30 min of Continuous rowing exercise at a heart rate intensity equivalent to 75% of VO2_Max_. After a rest period of at least 4 hours, subjects returned to complete lower body Resistance exercises. HIIT indicates high-intensity interval training. Each of the three HIIT protocols was performed once a week, and their order was randomly selected by week and counterbalanced by subject. The order of HIIT protocols was preserved for a given subject between VR conditions.

Five subjects completed a counterbalanced, within-subject study that examined the effect of our VR simulation on physiological and cognitive outcomes of a spaceflight-like exercise training scenario. Each subject first completed a 2-week SPRINT protocol using the M-MED, either with (VR condition) or without (No-VR condition) the VR simulation during the cardiovascular training sessions. After at least a wash out period of 1 month, each subject repeated the 2-week protocol in the opposite experimental condition. Two subjects started in the VR group, and three subjects started in the no-VR group. A timeline depicting a given subject’s 2-week protocol is depicted in Figure 3.

The VR condition was delivered *via* an HTC Vive Pro Eye (2018, HTC Corporation, New Taipei City, Taiwan). Subjects in the No-VR group could visually monitor their real-time heart rate through an iPad (2020, Apple Inc., Cupertino, CA) placed on the M-MED that ran the proprietary Polar app showing real-time heart rate. During the No-VR condition, subjects were not permitted to listen to audio devices during exercise.

### Outcome measures

A summary of the physiological and cognitive metrics analyzed and their schedule for the 2-week SPRINT protocol is given in Table 1. A more thorough explanation of each of the following metrics can be found in Keller et al. (2021), and a summary is provided below.

**TABLE 1:** Pre-post and daily physiological and cognitive metrics employed in the 2-week SPRINT protocol. The specific days when each measure was collected are also indicated in the table. Pre-post measures were collected before and after the 2-week SPRINT protocol. Daily measures were generally collected before and after each individual exercise session. Salivary cortisol samples were only collected prior to exercise sessions. Some other daily measures, indicated with an *, were collected only after exercise sessions.

Measure	Tool	Days used
***Pre-Post Measures***		
*Physiological*		
Maximal Oxygen Uptake	Stress Test	Day 0/14
Maximal Heart Rate	Stress Test	Day 0/14
Resting Energy Expenditure	Indirect Calorimetry	Day 0/14
Blood Pressure	Arm Cuff Sphygmomanometer	Day 0/14
Body Composition	DEXA & BMI	Day 0/14
Leg Muscular Strength	Leg Press	Day 0/14
Leg Muscular Power	Leg Press	Day 0/14
Leg Muscular Endurance	Leg Press	Day 0/14
*Cognitive*		
Virtual Reality Value	Value of Virtual Reality (Exercise)	Day 0/14
Emotional Distress	General Health Questionnaire (28 questions)	Day 0/14
Perceived Stress	Perceived Stress Scale (14 questions)	Day 0/14
Cognitive Function	WinSCAT	Day 0/14
Motivation	Sport Motivation Scale-6	Day 0/14
***Daily Measures***		
*Physiological*		
Physical Stress	Salivary Cortisol	Days 1,3,6,8,10,13
*Cognitive*		
Transient Anxiety	State-Trait Anxiety Inventory (T only)	Days 1–6, 8–13
State Feeling	Feeling Scale & Felt Arousal Scale	Days 1–6, 8–13
		Days 1–6, 8–13
Subjective Effort*	Rating of Perceived Exertion	Days 1–6, 8–13
Exercise Affect	Physical Activity Affect Scale	Days 1–6, 8–13
Mood Restoration*	Perceived Restorativeness Scale	Days 1–6, 8–13
Virtual Presence* (VR Only)	Spatial Presence Experience Scale	Days 1–6, 8–13

### Pre-post measures

A broad set of physiological and cognitive measures were collected before and after each of the 2-week SPRINT conditions (VR and No-VR conditions). Physiological measures included: maximal oxygen uptake (VO_2Max_) and maximal heart rate *via* stress test, resting energy expenditure (REE) *via* indirect calorimetry, resting blood pressure (systolic and diastolic) *via* arm cuff sphygmomanometer, body composition *via* dual-x-ray absorptiometry (DEXA), and leg muscular strength, power, and endurance *via* leg press. Cognitive measures included: VR value, a measure of the bias a person may have toward VR generally, *via* the Value of Virtual Reality questionnaire (adapted for exercise; (Anderson et al., 2017)), emotional distress *via* the General Health Questionnaire (Nagyova, 2005), perceived stress *via* the Perceived Stress Scale (Cohen et al., 1983), cognitive function *via* Windows Cognitive Aptitude Test (WinSCAT) (Kane and Kay, 1997), and motivation *via* Sport Motivation Scale-6 (SMS-6) (Mallett et al., 2007). The WinSCAT includes four sub-scales, each one delivered *via* its own software to test a specific cognitive function: 1) Code Memory, a test of short-term recall; 2) Running Memory, a test of sustained attention and concentration; 3) Match to Sample, a test of visual short-term memory; and 4) Mathematical Processing, a test of verbal working memory. The SMS-6 scale includes six sub-scales derived from 24 items that can be (simply) thought of as the spectrum of an individual’s motivation toward an exercise or sport, ranging from Amotivation (or a lack of motivation) to Intrinsic Motivation (or an in-born motivation independent of any external factors). A measurable transition from one sub-scale to an adjacent level or beyond over time represents an internalization or internal reorganization of the various motivational factors.

### Daily measures

Another set of physiological and psychological measures was collected before and/or after each individual exercise session. To measure physical stress, two salivary cortisol samples were simultaneously collected prior to the exercise sessions on protocol days 1, 3, 6, 8, 10, and 13. Daily, cognitive metrics included transient anxiety *via* a short-form of the State-Trait Anxiety Inventory (Marteau and Bekker, 1992), state feeling regarding exercise *via* the Feeling Scale (Hardy and Rejeski, 2016) and the Felt Arousal Scale (Svebak and Murgatroyd, 1985), subjective effort *via* the Rating of Perceived Exertion (RPE) questionnaire (Borg, 1962), exercise affect *via* the Physical Activity Affect Scale (PAAS) (Lox et al., 2000), and mood restoration *via* the Perceived Restorativeness Scale (PRS) (Hartig et al., 1997). The PRS questionnaire includes four sub-scales described succinctly as: 1) *compatibility*, the feeling that one’s goals and intentions are matched to the environment’s capacity to allow them to achieve those goals; 2) *being away*, the feeling of escaping unwanted distractions external to the activity’s context; 3) *fascination*, the feeling of being able to direct attention effortlessly toward contents and events in the environment; and 4) *coherence*, the feeling that environment is a calm and predictable place. Additionally, for the VR group only, virtual presence, commonly used as a metric of the immersive qualities of a VR simulation, was also measured *via* the Spatial Presence Experience Scale (SPES) (Hartmann et al., 2016). These cognitive metrics were collected before and after each individual exercise session, except for subjective effort, mood restoration, and virtual presence, which were collected after exercise sessions only.

### Statistical analysis

Much of the data did not satisfy the normality assumption, most likely due to the low number of subjects. Thus, non-parametric statistical techniques were implemented.

Pre-Post-measures were examined using paired samples Wilcoxon rank tests to investigate the effects of *time* (i.e., post vs. pre) and *VR condition* ( $∆\left(VR\right)={Post}_{VR}-{Pre}_{VR}$ vs $∆\left(No\;VR\right)={Post}_{No\;VR}-{Pre}_{No\;VR}$ ).

Daily measures and salivary cortisol were analyzed *via* a three-way, aligned-rank transform (ART) repeated measures analyses of variance to determine factor and interaction effects (Wobbrock et al., 2011). Factors studied includes *VR condition* (VR vs No-VR), *time*, and *workout* (HIIT vs Continuous 30 min of exercise). When measures were collected before and after an exercise session (i.e., transient anxiety, state feeling, and exercise affect), the delta between the two ( ∆=Post−Pre ) was considered the metric of interest.

Data are presented as mean ± SE. A two-sided alpha level of 0.05 was chosen *a priori* for all statistical tests. Statistics were conducted using R Version 4.1.0 (2022, R Foundation for Statistical Computing, Vienna, Austria)*.*

## Results

### Pre-post measures

Table 2 summarizes the results of the pre-post physiological and cognitive measures. Effects of the 2-week protocol on pre-post physiological measures were mostly similar within and between the VR and No-VR groups throughout the protocol. ∆ VO_2Max_ was significantly higher in the No-VR group with respect to the VR group (*p* = 0.043). Leg press strength increased over time across both groups (VR: *p* = 0.042, No-VR: *p* = 0.043). A significant effect of time indicated increased muscular power in the No-VR group (*p* = 0.043). No other significant differences were detected for *time* or *VR condition* for maximal heart rate, REE, blood pressure, or body composition.

**TABLE 2:** Summary of pre-post measures collected before and after the 2-week SPRINT protocol for the VR and No-VR groups (n = 5). Data were analyzed using paired sample Wilcoxon rank tests to investigate the effects of time (post v. pre) and VR condition (VR condition ( $∆\left(VR\right)={Post}_{VR}-{Pre}_{VR}$ vs $∆\left(No\;VR\right)={Post}_{No\;VR}-{Pre}_{No\;VR}$ ). Data are presented as mean ± SE. Bolded items indicate *p* < 0.05.

Pre/Post measures	—	Pre	Post	Δ = post-pre	*p* Value			
					Time	VR
**Physiological**						
Maximal Oxygen Uptake (ml/kg/min)	VR	34.6 ± 2.3	34.6 ± 3.3	0.0 ± 1.0	0.225	**0.043**
	No-VR	35.3 ± 2.1	37.6 ± 3.4	2.3 ± 1.3	0.893		
Maximal Heart Rate (bpm)	VR	180 ± 2.5	172 ± 2.9	−8 ± 0.4	0.345	0.581
	No-VR	184 ± 4.1	174 ± 0.5	−10 ± 3.6	0.104		
Resting Energy Expenditure (kcal)	VR	1918 ± 183	1896 ± 152	−21.5 ± 31.0	0.225	0.225
	No-VR	1894 ± 180	1970 ± 235	76 ± 54.6	0.686		
Resting Systolic Blood Pressure (mmHg)	VR	119 ± 3.2	116 ± 3.7	−3.2 ± 0.5	0.356	0.138
	No-VR	118 ± 5.9	123 ± 6.1	5.2 ± 0.2	0.363		
Resting Diastolic Blood Pressure (mmHg)	VR	76 ± 2.4	74 ± 2.6	−1.4 ± 0.1	0.525	0.465
	No-VR	75 ± 3.8	78 ± 3.7	2.8 ± 0.1	0.418		
Body Fat (%)	VR	22.6 ± 3.4	22.0 ± 3.8	−0.7 ± 0.4	0.893	0.136
	No-VR	22.2 ± 3.5	22.5 ± 3.5	0.2 ± 0.0	0.225		
Leg Strength (kg)	VR	427 ± 56	461 ± 57	33.6 ± 0.7	**0.042**	0.500
	No-VR	462 ± 58	481 ± 58	19.0 ± 0.3	**0.043**		
Leg Power (W)	VR	1915 ± 260	2011 ± 292	95.6 ± 31.8	0.225	0.686
	No-VR	1910 ± 255	1995 ± 278	84.6 ± 22.7	**0.043**		
Leg Endurance (W)	VR	973 ± 132	1011 ± 157	38.2 ± 24.2	0.893	0.686
	No-VR	972 ± 166	985 ± 159	13.0 ± 7.0	0.345		
**Cognitive**						
Value of Virtual Reality (Exercise)	VR	26.0 ± 2.1	31.8 ± 3.2	5.8 ± 1.1	0.068	0.893
	No-VR	28.8 ± 4.2	34.4 ± 2.6	5.6 ± 1.6	0.104		
General Health Questionnaire	VR	44.8 ± 1.6	47.2 ± 2.6	2.4 ± 1.0	0.416	0.893
	No-VR	43.2 ± 1.5	44.4 ± 2.4	1.2 ± 0.9	0.715		
Perceived Stress Scale	VR	32.2 ± 3.5	34.8 ± 3.6	2.6 ± 0.1	0.465	0.715
	No-VR	33.4 ± 4.2	35.6 ± 4.7	2.2 ± 0.5	0.465		
*WinSCAT Sub-scales*
Code Memory
*Reaction Time (ms)*	VR	913 ± 91	979 ± 92	66.8 ± 1.0	0.500	**0.043**
	No-VR	1028 ± 116	993 ± 116	−34.4 ± 0.0	0.686		
*Accuracy (%)*	VR	95.4 ± 2.1	97.6 ± 1.5	2.2 ± 0.6	0.357	0.197
	No-VR	97.6 ± 1.7	94.4 ± 2.5	−3.2 ± 1.0	0.317		
Running Memory						
*Reaction Time (ms)*	VR	534 ± 54	580 ± 40	45.4 ± 14.4	0.465	0.225
	No-VR	561 ± 44	572 ± 51	10.2 ± 7.8	0.080		
*Accuracy (%)*	VR	85.8 ± 9.5	84.0 ± 8.8	−1.8 ± 0.7	1.000	0.336
	No-VR	92.2 ± 2.6	92.4 ± 1.2	0.2 ± 1.4	0.416		
*Losses*	VR	19.6 ± 15.7	21.4 ± 15.1	1.8 ± 0.6	0.684	0.465
	No-VR	7.6 ± 3.7	5.8 ± 1.4	−1.8 ± 2.7	0.684		
Match to Sample						
*Reaction Time (ms)*	VR	1466 ± 136	1713 ± 126	246.8 ± 10.8	0.893	**0.043**
	No-VR	1439 ± 69	1462 ± 94	23.2 ± 24.3	**0.043**		
*Accuracy (%)*	VR	98.6 ± 1.4	97.2 ± 1.7	−1.4 ± 0.3	0.581	0.357
	No-VR	96 ± 2.6	98.2 ± 1.4	2.2 ± 1.3	0.564		
Mathematical Processing						
*Reaction Time (ms)*	VR	2204 ± 286	2184 ± 303	−20.4 ± 17.0	0.893	0.686
	No-VR	2170 ± 359	2175 ± 250	4.2 ± 108.9	0.500		
*Accuracy (%)*	VR	89.0 ± 1.0	91.0 ± 2.9	2.0 ± 1.9	0.066	0.222
	No-VR	96.0 ± 1.8	73.0 ± 16.3	-23.0 ± 14.4	0.480		
*Sport Motivation Scale Sub-Scales*						
Amotivation	VR	7.8 ± 3.3	4.4 ± 0.2	−3.4 ± 3.1	0.109	0.109
	No-VR	5.8 ± 1.9	7.4 ± 2.7	1.6 ± 0.8	0.257		
External Regulation	VR	9.8 ± 2.5	12.8 ± 4.80	3.0 ± 2.2	0.581	0.893
	No-VR	12.6 ± 4.5	13.0 ± 5.0	0.4 ± 0.5	1.000		
Introjected Regulation	VR	16.8 ± 2.5	18.2 ± 2.7	1.4 ± 0.3	0.461	0.786
	No-VR	14.8 ± 2.2	16.6 ± 2.0	1.8 ± 0.1	0.893		
Identified Regulation	VR	16.6 ± 3.4	18.2 ± 2.7	0.4 ± 0.9	0.917	0.345
	No-VR	14.6 ± 2.6	17.0 ± 3.1	2.4 ± 0.5	0.080		
Integrated Regulation	VR	15.0 ± 3.6	17.8 ± 3.7	2.8 ± 0.1	0.593	1.000
	No-VR	14.8 ± 8.7	15.4 ± 10.0	0.6 ± 2.2	1.000		
Intrinsic Motivation	VR	19 ± 3.9	22.0 ± 2.9	3.0 ± 1.0	0.273	1.000
	No-VR	19.8 ± 3.1	21.8 ± 2.9	2.0 ± 0.2	0.588		

**FIGURE 4:**
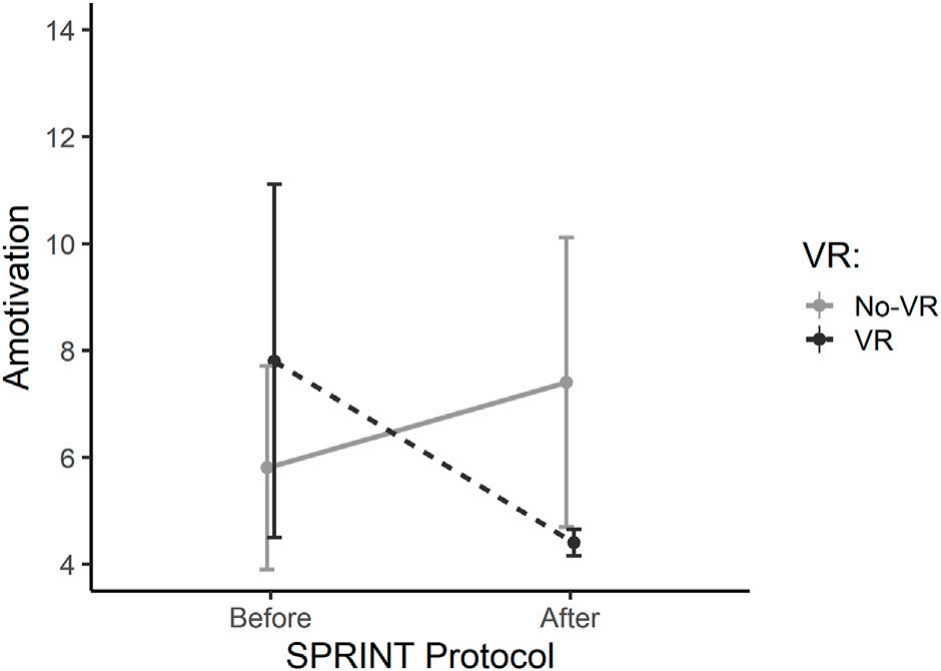
Sport Motivation Scale-6 (SMS-6) sub-scale: Amotivation, before and after the 2-week SPRINT exercise protocol for the VR (dark grey, dashed bar) and No-VR groups (light grey, solid bar) (n = 5). Results show mild trends of decreasing Amotivation for the VR group over time (*p* = 0.109). Overall differences between VR and No-VR condition in this subscale were mild (*p* = 0.109).

Results of the WinSCAT testing showed that Code Memory reaction time increased (not significantly) for the VR group and decreased (not significantly) for the No-VR group, and these pre-post changes between VR groups were statistically different (*p* = 0.043). In addition, both VR groups became slower in the Match to Sample reaction time metric, but only the No-VR group reaction time significantly increased (*p* = 0.043). These pre-post changes in Match to Sample reaction time between VR groups were also statistically different (*p* = 0.043). Mathematical Processing accuracy trended upward over time in the VR group (*p* = 0.066). The Sport Motivation subscale Amotivation is shown in Figure 4. Results show mild trends of decreasing Amotivation for the VR group (*p* = 0.109). Overall differences between VR and No-VR condition in this subscale were mild (*p* = 0.109). No other metrics of cognition approached significance.

### Daily measures

**FIGURE 5:**
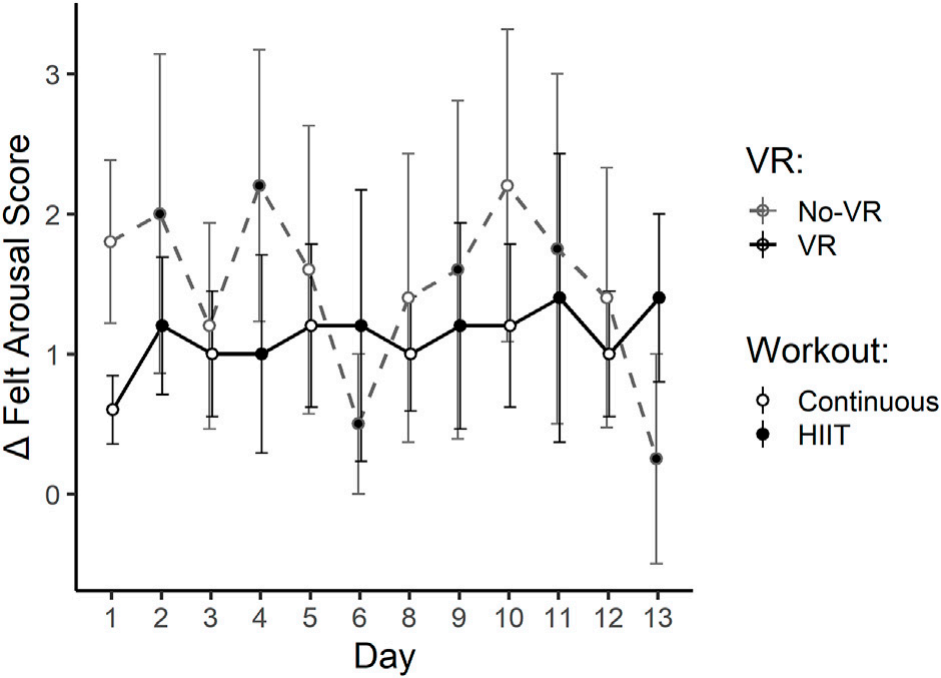
Daily scores for ∆ Felt Arousal collected before and after each individual exercise session ( ∆=(Post−Pre) ) throughout the 2-week SPRINT exercise protocol, for the VR group (black, solid line) and No-VR group (grey, dashed line) (n = 5). Open symbols indicate 30-min of continuous workouts (rowing exercise at a heart rate intensity equivalent to 75% of VO2_Max_), and closed symbols indicate high-intensity interval training (HIIT) workouts. Statistical testing did not show a significant effect of *VR Condition*. However, there was a significant interaction between *VR Condition x Time* (*p* = 0.039), indicating decreasing Felt Arousal Scores in the No-VR group over time. Data are presented as mean ± SE.

Table 3 summarizes the results of the daily measures. Significant physical stress changes over time, between VR groups, or between workouts as determined by salivary cortisol could not be determined. Felt Arousal was not found to be significantly different with respect to the three main factors investigated (*VR Condition*, *Time*, and *Workout*), although the *VR Condition* × *Time* interaction was significant (*p* = 0.039), showing decreasing scores in the No-VR group (see Figure 5).

**TABLE 3:** Summary of daily measures collected before and/or after each individual exercise session, for the VR and No-VR groups (n = 5). Salivary cortisol samples were simultaneously collected prior to the exercise sessions on protocol days 1, 3, 6, 8, 10, and 13. Rating of Perceived Exertion (RPE), Perceived Restoratives sub-Scales (PRS), and the Spatial Presence Experience Scale (SPES) were collected after the exercise sessions only (measures indicated with †). The rest of the scales were collected before and after each individual exercise session and these metrics are presented as delta ∆=Post−Pre . Data were analyzed using a three-way, aligned-rank transform (ART) repeated measures analyses of variance with factors *VR Condition* (VR vs No-VR), *Time*, and *Workout* (HIIT vs Continuous, with HIIT training occurring on days indicated by shaded columns). Significance for main effects and interaction effects are reported. Data are presented as mean ± SE. Bolded items indicate *p* < 0.05. Italicized items indicate 0.05 < *p* < 0.1.

Daily exercise effects of VR	Group	Protocol day												*p*-value				
		1	2	3	4	5	6	8	9	10	11	12	13	VR	Time	Workout	VR x workout	VR x time
Salivary Cortisol (ng/ml)	VR	48.9 ± 12.2	—	29.4 ± 5.3	—	—	35.7 ± 6.9	32.8 ± 2.1	—	48.8 ± 8.2	—	—	28.5 ± 5.4	0.193	0.114	0.947	0.738	0.776
	No-VR	53.2 ± 7.0	—	45.0 ± 12.4	—	—	42.9 ± 12.1	31.9 ± 2.6	—	63.4 ± 13.6	—	—	51.6 ± 13.2					
Δ State Trait Anxiety Score	VR	2.4 ± 1.6	1.2 ± 1.6	−0.2 ± 0.9	1.4 ± 1.2	−0.2 ± 0.9	1.0 ± 1.2	1.0 ± 0.6	−0.2 ± 1.5	1 ± 1.0	−0.4 ± 1.1	0.8 ± 0.6	1.6 ± 1.6	0.283	0.176	0.756	0.107	0.995
	No-VR	0.2 ± 1.2	1.4 ± 1.4	−1.4 ± 1.6	2.0 ± 1.1	−0.8 ± 1.0	2.5 ± 1.2	1.4 ± 1.8	0.0 ± 1.0	−1.6 ± 1.8	−0.5 ± 0.7	−0.6 ± 1.0	3.5 ± 1.6					
Δ Feeling Score	VR	0.4 ± 0.4	1.8 ± 0.4	1.6 ± 0.6	0.8 ± 0.6	1.2 ± 0.5	0.8 ± 0.6	0.8 ± 0.8	1.2 ± 0.8	0.8 ± 0.8	1.2 ± 0.5	1.0 ± 0.5	1.0 ± 0.5	0.897	0.282	0.593	0.887	0.868
	No-VR	1.6 ± 0.9	1.0 ± 0.8	1.8 ± 0.8	1.4 ± 0.7	1.6 ± 0.7	−0.5 ± 0.7	0.8 ± 1.1	1.4 ± 1.2	2.6 ± 1.2	1.5 ± 1.0	1.8 ± 0.8	−0.8 ± 1.2					
Δ Felt Arousal Score	VR	0.6 ± 0.2	1.2 ± 0.5	1.0 ± 0.5	1.0 ± 0.7	1.2 ± 0.6	1.2 ± 1.1	1.0 ± 0.4	1.2 ± 0.7	1.2 ± 0.6	1.4 ± 1.0	1.0 ± 0.5	1.4 ± 0.6	0.853	0.951	0.772	0.886	**0.039**
	No-VR	1.8 ± 0.6	2.0 ± 1.1	1.2 ± 0.7	2.2 ± 1.0	1.6 ± 1.0	0.5 ± 0.5	1.4 ± 1.0	1.6 ± 1.2	2.2 ± 1.1	1.8 ± 1.3	1.4 ± 0.9	0.3 ± 0.8					
Rating of Perceived Exertion (RPE)†	VR	15.2 ± 0.4	16.4 ± 0.2	15.0 ± 0.3	17.0 ± 0.6	15.0 ± 0.3	17.0 ± 0.5	14.3 ± 0.4	16.4 ± 0.5	14.8 ± 0.7	17.8 ± 0.4	15.2 ± 0.4	16.6 ± 0.7	0.155	0.962	**0.001**	0.528	0.540
	No-VR	15.2 ± 0.4	15.2 ± 0.4	15.4 ± 0.8	17.8 ± 0.5	15.0 ± 0.5	16.3 ± 1.4	15.2 ± 0.6	16.6 ± 0.4	12.4 ± 3.1	17.5 ± 0.3	15.0 ± 0.6	17.3 ± 0.6					
Physical Activity Affect Score Sub-scales																		
*Δ Positive Affect*	VR	0.2 ± 0.7	2.4 ± 0.9	2.4 ± 1.0	1.8 ± 1.2	0.6 ± 0.5	1.0 ± 1.3	1.0 ± 0.5	2.2 ± 0.7	2.2 ± 0.7	0.8 ± 0.7	0.6 ± 0.7	2.2 ± 1.0	0.429	*0.099*	0.661	0.363	0.292
	No-VR	2.8 ± 0.9	2.4 ± 1.1	1.8 ± 0.6	1.6 ± 1.2	1.6 ± 1.2	−1.0 ± 0.9	2.6 ± 1.4	2.0 ± 1.1	4.0 ± 1.4	2.25 ± 2.6	1.6 ± 1.4	−1.0 ± 2.6					
*Δ Negative Affect*	VR	−0.2 ± 0.2	−0.2 ± 0.2	0.0 ± 0.0	0.0 ± 0.0	0.0 ± 0.0	0.0 ± 0.0	−1.0 ± 0.9	−0.4 ± 0.5	0.0 ± 0.0	0.0 ± 0.0	0.0 ± 0.0	0.2 ± 0.2	*0.083*	0.506	*0.087*	**0.026**	**0.009**
	No-VR	−0.6 ± 0.9	0.6 ± 0.4	−1.2 ± 0.8	0.4 ± 0.5	−0.2 ± 0.3	−0.25 ± 0.3	0.2 ± 0.2	0.2 ± 0.2	0.0 ± 0.0	0.5 ± 0.5	−0.2 ± 0.2	1.0 ± 0.7					
*Δ Fatigue*	VR	2.8 ± 1.7	1.4 ± 1.2	−0.4 ± 0.4	0.4 ± 0.2	0.8 ± 0.6	2.4 ± 1.4	0.3 ± 0.2	0.8 ± 0.6	1.4 ± 0.7	1.6 ± 0.8	0.6 ± 0.7	0.8 ± 0.7	*0.076*	0.591	0.491	**0.015**	0.134
	No-VR	0.4 ± 1.0	2.4 ± 1.7	1.2 ± 1.2	2.0 ± 0.6	0.8 ± 0.9	2.0 ± 1.1	1.0 ± 0.8	1.2 ± 0.8	1.0 ± 0.3	2.25 ± 1.0	0.4 ± 0.4	3.5 ± 0.9					
*Δ Tranquility*	VR	−1.4 ± 1.8	−1.0 ± 0.6	0.0 ± 0.6	−0.6 ± 0.9	0.2 ± 0.7	0.2 ± 1.7	−1.0 ± 1.0	−1.4 ± 0.8	−1.2 ± 1.0	0.0 ± 1.1	−1.0 ± 1.3	−2.0 ± 1.4	0.216	0.823	0.794	0.690	0.125
	No-VR	−1.2 ± 0.9	−1.2 ± 1.2	0.4 ± 1.1	−1.6 ± 0.8	0.8 ± 0.7	0.3 ± 0.6	−1.0 ± 1.4	0.6 ± 0.9	1.2 ± 2.1	0.8 ± 0.8	0.2 ± 0.6	0.0 ± 1.4					
Perceived Restorativeness Sub-scales (PRS)†																		
*Compatibility*	VR	38.4 ± 8.4	38.0 ± 8.1	40.8 ± 8.0	42.2 ± 9.0	39.8 ± 8.5	41.6 ± 8.6	37.8 ± 8.3	43.0 ± 7.5	44.6 ± 8.0	42.6 ± 8.2	44.2 ± 8.1	46.6 ± 7.0	**0.050**	0.823	0.794	0.346	0.527
	No-VR	36.8 ± 4.5	35.0 ± 4.9	39.4 ± 4.8	36.2 ± 4.7	33.8 ± 5.3	29.3 ± 3.8	36.4 ± 5.7	35.2 ± 6.0	30.4 ± 9.5	39.0 ± 6.5	38.2 ± 5.8	35.0 ± 4.3					
*Being Away*	VR	23.4 ± 3.6	24.0 ± 4.1	23.6 ± 4.3	24.6 ± 5.3	25.2 ± 4.9	25.8 ± 5.3	22.0 ± 5.3	26.2 ± 4.4	26.8 ± 4.2	26.2 ± 4.4	26.4 ± 4.5	26.2 ± 4.6	0.114	0.942	0.832	0.686	0.181
	No-VR	26.2 ± 3.4	26.2 ± 3.5	24.2 ± 3.7	23.4 ± 4.2	24.4 ± 3.9	21.75 ± 5.0	23.4 ± 4.0	23.0 ± 4.5	16.4 ± 5.3	20.5 ± 4.6	23.8 ± 4.5	19.5 ± 4.9					
*Fascination*	VR	37.2 ± 5.2	38.0 ± 5.9	38.0 ± 6.4	37.6 ± 7.5	36.8 ± 7.7	35.4 ± 7.1	33.5 ± 7.4	38.6 ± 7.4	37.4 ± 7.0	38.4 ± 7.3	37.6 ± 7.2	39.0 ± 8.2	**< 0.001**	0.959	0.966	0.905	0.991
	No-VR	28.8 ± 3.8	27.4 ± 4.6	30.0 ± 6.1	27.4 ± 5.5	26.8 ± 6.1	23.8 ± 4.9	26.2 ± 5.7	26.8 ± 6.2	20.4 ± 8.4	27.0 ± 9.6	28.6 ± 6.3	24.3 ± 5.7					
*Coherence*	VR	27.2 ± 0.8	28.0 ± 0.0	28.0 ± 0.0	27.8 ± 0.2	27.8 ± 0.2	27.6 ± 0.5	27.8 ± 0.2	27.8 ± 0.2	27.6 ± 0.4	27.8 ± 0.2	27.6 ± 0.4	27.8 ± 0.2	**< 0.001**	0.264	0.385	0.292	0.130
	No-VR	25.6 ± 1.7	25.6 ± 1.6	25.6 ± 1.6	25.0 ± 1.9	24.8 ± 1.9	26.0 ± 1.7	25.2 ± 2.6	25.2 ± 2.6	19.4 ± 5.1	24.0 ± 2.5	25.4 ± 1.9	25.3 ± 2.8					
Spatial Presence Experience Sub-Scales (SPES)†																		
*Self-Location*	VR	10.6 ± 0.6	11 ± 0.6	10.2 ± 1.2	10.0 ± 1.3	10.0 ± 1.3	10.0 ± 1.3	9.75 ± 1.5	10.0 ± 1.3	10.6 ± 1.2	10.4 ± 1.2	10.4 ± 1.2	10.6 ± 1.0	N/A	0.614	0.999	N/A	N/A
*Possible Actions*	VR	9.0 ± 0.9	9.4 ± 1.2	9.6 ± 1.3	9.0 ± 1.3	9.4 ± 1.2	10.0 ± 1.5	9.5 ± 1.4	8.2 ± 1.6	9.4 ± 1.4	9.2 ± 1.5	9.6 ± 1.5	9.6 ± 1.4	N/A	0.628	0.999	N/A	N/A

Concerning subjective effort, an overall effect of *Workout* indicated that HIIT training elicited significantly higher RPE scores than Continuous training (*p* < 0.001), independently of the *VR Condition* or *Time*.

**FIGURE 6:**
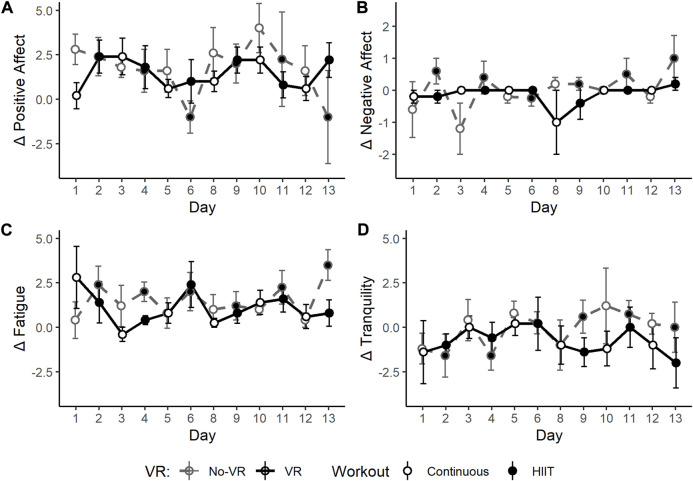
Daily scores for the Physical Activity Affect Scale (PAAS) sub-scales: ∆ Positive Affect **(A)**, ∆ Negative Affect **(B)**, ∆ Fatigue **(C)**, and ∆ Tranquility **(D)**, collected before and after each individual exercise session ( ∆=(Post−Pre) ) throughout the 2-week SPRINT exercise protocol, for the VR group (black, solid line) and No-VR group (grey, dashed line) (n = 5). Open symbols indicate 30-min of continuous workouts (rowing exercise at a heart rate intensity equivalent to 75% of VO2_Max_)_,_ and closed symbols indicate high-intensity interval training (HIIT) workouts. While not statistically significant, ∆ Negative Affect and ∆ Fatigue were generally higher in the No-VR group with respect to the VR group (Δ Negative Affect *p* = 0.083; *Δ* Fatigue *p* = 0.076). For *Δ* Negative Affect, the interaction effects between *VR Condition* x *Time* were significant for the No-VR group (*p* = 0.009), which showed an increase in *Δ* Negative Affect over time. In addition, interaction effects of *VR Condition* x *Workout* were also significant for *Δ* Negative Affect (*p* = 0.026) and *Δ* Fatigue (*p* = 0.015), indicating significantly higher No-VR scores on HIIT training days. Data are presented as mean ± SE.

Physical Activity Affect Scale (PAAS) subscales, shown in Figure 6, did not revealed significant overall effects of *VR Condition*, *Time*, or *Workout*, although the delta ((post–pre) exercise session) for Negative Affect and Fatigue were generally higher in the No-VR group with respect to the VR group (*Δ* Negative Affect *p* = 0.083; *Δ* Fatigue *p* = 0.076). In the No-VR group, the interaction of *VR Condition* and *Time* was significant for *Δ* Negative Affect (*p* = 0.009), which over time, generally increased compared to the VR group. In addition, *Δ* Negative Affect (*p* = 0.026) and *Δ* Fatigue (*p* = 0.015) also showed a significant *VR Condition* × *Workout* interaction effect, indicating significantly higher No-VR scores during HIIT training.

**FIGURE 7:**
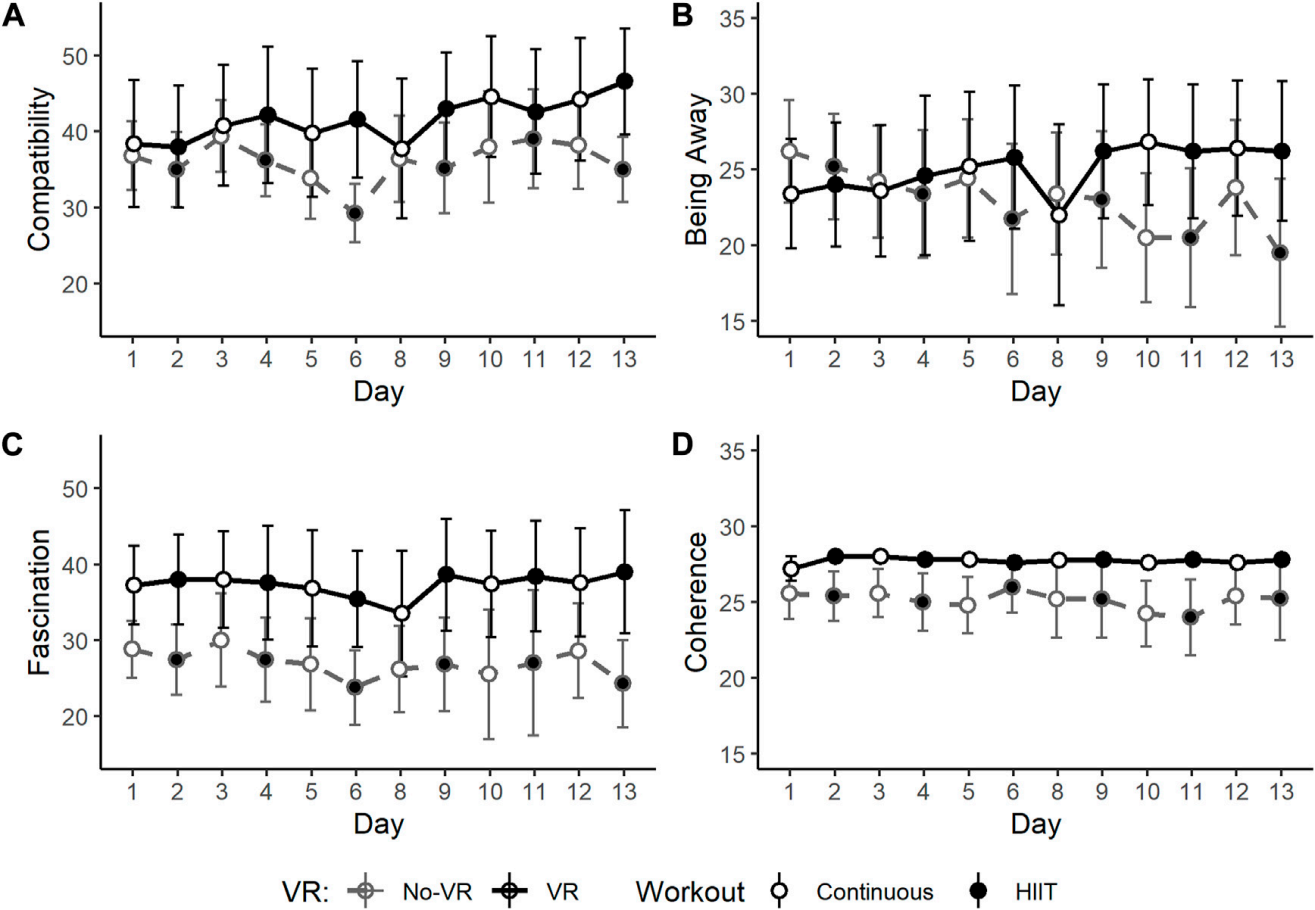
Daily scores for Perceived Restorativeness Scale (PRS) sub-scales: Compatibility **(A)**, Being Away **(B)**, Fascination **(C)**, and Coherence **(D)**, collected daily after each individual exercise session throughout the 2-week SPRINT exercise protocol, for the VR group (black, solid line) and No-VR group (grey, dashed line) (n = 5). Open symbols indicate 30-min of continuous workouts (rowing exercise at a heart rate intensity equivalent to 75% of VO2_Max_)_,_ and closed symbols indicate high-intensity interval training (HIIT) workouts. Fascination and Coherence scores were statistically higher in the VR group compared to the No-VR group (*p* < 0.001 for both). Compatibility scores were also marginally significantly higher in the VR group compared to the No-VR group (*p* = 0.050). Data are presented as mean ± SE.

Perceived Restorativeness Scale (PRS) sub-scales, shown in Figure 7, showed significantly higher scores in the VR group compared to the No-VR group in two of the four subscales: Fascination (*p* < 0.001) and Coherence (*p* < 0.001). Scores of the sub-scale Compatibility scores in the VR condition were also higher than in the No- VR condition, but they were just marginally significant (*p* = 0.050). 

Overall means for Spatial Presence subscales (VR group only) for Self-Location (SL) (10.3 ± 0.36) and Possible Actions (PA) (9.3 ± 0.45) remained stable over time (*p* = 0.614, and 0.628, respectively), and were not detectably different between the Continuous (SL = 2.2 ± 0.34; PA = 2.54 ± 0.022) and HIIT (SL = 2.24 ± 0.41; PA = 2.78 ± 0.61) training days (SL *p* = 0.999; PA *p* = 0.999).

## Discussion

This pilot study analyzed the effect of VR exergaming on physiological and cognitive performance metrics of a male astronaut-like population during a 2-week, spaceflight-validated, exercise countermeasure protocol on a prototype exercise device designed for the space environment. The successful deployment and integration of the VR condition demonstrated interesting trends in cognitive measures. These included improvements in metrics of mood restoration and exercise affect. Positive trends were also seen in mathematical processing accuracy, visual short-term memory (Match to Sample) reaction times, felt arousal, and reduced amotivation when compared to the identical exercise protocol in the same subjects without VR. However, trends in VO_2Max_ and in metrics of short-term recall (Code Memory) suggest that the VR condition may not have improved performance as much as the No-VR condition. Physiological effects of *Time* and *VR Condition* were minimal, but this was not unexpected given the short duration of the study.

These pilot results support growing evidence for the efficacy of VR in exercise performance outcomes (De La Torre et al., 2018). VR exergaming is an emerging field in recreational activity (Finkelstein et al., 2010) and rehabilitation (Asadzadeh et al., 2021), with promising outcomes in physical as well as mental and social health (Loos and Kaufman, 2018). Strong SPES sub-scale scores indicate high immersive qualities of the custom VR condition created for this pilot study. In a 2017 systematic review, Matallaoui et al. showed that few exergaming studies integrated gamification design principles, favoring instead a basic shift from button-based inputs to movement-based inputs alone, and that effects of exergaming could be enhanced if those design principles were considered (Matallaoui et al., 2017). The design implemented in the present study includes several such principles: an in-game avatar, continual progress toward a known goal, and virtual competition. Previously established restorative effects of exercise, particularly when considered in light of the isolation and confinement inherent to long-duration spaceflight simulations and the COVID-19 pandemic, have been further elevated with the integration of VR (Choukér and Stahn, 2020; van Cutsem et al., 2022). Trends seen here in amotivation, felt arousal, physical activity affect, and restorativeness suggest that the VR condition generally augments the effects of exercise alone on these metrics, a finding that supports a 2021 systematic review on the use of VR in exercise rehabilitation (Asadzadeh et al., 2021). Further, the slope of trends seen in Figures 4–7 suggest that a longer-term study with more subjects may yield more significant results, like those seen in a previous VR vs No-VR running study (Neumann and Moffitt, 2018). It is also possible that some subjects found the presence of a competing boat to be a motivational factor, even if the boat was virtual, an effect also seen previously in VR (Murray et al., 2016; Parton and Neumann, 2019). VR alone (absent exercise) has been demonstrated to elicit restorative effects (Anderson et al., 2017), therefore the possibility of enhancing this effect with exercise seems worthy of further investigation.

It is particularly noteworthy that despite the small sample size, clear trends emerged in the efficacy of the VR condition in the more intense modes of exercise. The SPRINT protocol mandates high-intensity interval training on alternating days, and it was on these days (closed symbols in Figures 5–7) that many of the strongest differences manifested between the VR and No-VR groups, such as lower negative affect and fatigue. This is similar to other findings of VR effects in high-intensity modes (Barathi et al., 2018; Farrow et al., 2019).

These findings are also relevant to the burgeoning industry of exergaming. To tackle the issue rising global obesity, or even to provide viable ways of escaping isolation and confinement in a future pandemic, strategies for making physical activity more accessible, engaging, and rewarding may be possible through VR. To our knowledge, no market solution currently available integrates real-time biometric data with exergaming performance such as what was studied here. This gap represents a possible innovation that merits further investigation. Future work should include alternate natural landscapes for users to choose from, and additional, optional, competitive elements such as competing against personal records and/or against the performance of other users.

### Limitations

While the M-MED is a robust and versatile platform for exactly this kind of study, it was never designed to be flown to space and therefore lacks many of the mechanical constraints required by later, more modern, designs such as the MED-2 (Downs et al., 2017). The novel nature of the hardware integration pipeline from chest-strap monitor, to bespoke software integration, proprietary software integration, and finally wired headset display, suffered from reliability and ergometric setbacks. Namely, the setup and implementation of the data stream could be simplified, and the VR headset would greatly benefit from a wireless adapter to prevent the cord from interrupting user movement. The VR simulation itself, while very promising, was limited to a single river competition setting for use in the rowing configuration of the M-MED.

While metrics of exercise performance and body composition were examined, no specific muscles were analyzed. Long-term unloading of weight-bearing muscles elicits pronounced atrophy, as noted in the introduction, and exercise countermeasures to this effect should ensure these muscles are protected.

Finally, it is clear that the study could benefit from greater statistical power. However, initial human testing started in the spring of 2020 halted due to the global COVID-19 pandemic. Budgetary constraints prevented the complete replacement of previous data, which therefore precluded many of the correlative and modeling calculations we had planned to run on sub-groups such as VR biases and personality types. It should also be noted that subjects who began in the VR group only to repeat the protocol later without VR may have experienced attitudes toward the protocol, which were not captured, nor offset, by any attitude changes in subjects who started without VR. In effect, a hypothetically disappointed subject’s cognitive scores may not have been offset by an excited subject’s cognitive scores who received the VR condition in the opposite order. A larger study will need to be conducted to detect such an effect, if it exists.

## Conclusion

The intervention of real-time biometrically-integrated VR with spaceflight exercise protocols was sufficient to elicit detectable differences between VR and No-VR groups in VO2_Max_ and several cognitive metrics. Spatial Presence Experience Scale (SPES) scores of the VR environment began high and maintained these performance values throughout the 2-week protocol, indicating a strong immersive quality into the VR scenario. Differences in outcomes generally favored the VR condition, including activity Negative Affect, Fatigue, Compatibility, Fascination and Coherence, although VO2_Max_ and some cognitive measures (Code Memory, and Match-to-Sample reaction times) were more favorable in the No-VR group. The VR condition showed further differences in cognitive metrics of Negative Affect, Fatigue, and Being Away when comparing high-to moderate-intensity cardiovascular workouts. Further trends in motivation subscales, exercise affect subscales, felt arousal, and restorativeness subscales were noteworthy but not significantly different in this pilot study. Carrying on this work with a longer timeline and in more limiting environments like those enforced during social lockdowns, long-term bedrest, or space exploration, may demonstrate more robust effects. These conclusions merit a more thorough evaluation through future studies of long-term isolation and confinement interventions, as well as investigations into the effects of VR exergaming on exercise outcomes in the general population.

## Data Availability

The raw data supporting the conclusions of this article will be made available by the authors, without undue reservation.
